# Bayesian estimation of the effect of health inequality in disease detection

**DOI:** 10.1186/s12939-022-01713-5

**Published:** 2022-08-27

**Authors:** Dinah Jane Lope, Haydar Demirhan, Anil Dolgun

**Affiliations:** School of Science, Mathematical Sciences Discipline, RMIT University, Melbourne, 3000 Australia

**Keywords:** Gini coefficient, Lorenz curve, Bayesian statistics, Bayesian model averaging, Influenza, Case detection, Incidence

## Abstract

**Background:**

Measuring health inequality is essential to ensure that everyone has equal accessibility to health care. Studies in the past have continuously presented and showed areas or groups of people affected by various inequality in accessing the health resources and services to help improve this matter. Alongside, disease prevention is as important to minimise the disease burden and improve health and quality of life. These aspects are interlinked and greatly contributes to one’s health.

**Method:**

In this study, the Gini coefficient and Lorenz curve are used to give an indication of the overall health inequality. The impact of this inequality in granular level is demonstrated using Bayesian estimation for disease detection. The Bayesian estimation used a two-component modelling approach that separates the case detection process and incidence rate using a mixed Poisson distribution while capturing underlying spatio-temporal characteristics. Bayesian model averaging is used in conjunction with the two-component modelling approach to improve the accuracy of estimates by incorporating many candidate models into the analysis instead of using fixed component models. This method is applied to an infectious disease, influenza, in Victoria, Australia between 2013 and 2016 and the corresponding primary health care of the state.

**Result:**

There is a relatively equal distribution of health resources and services pertaining to general practitioners (GP) and GP clinics in Victoria, Australia. Roughly 80 percent of the population shares 70 percent of the number of GPs and GP clinics. The Bayesian estimation with model averaging revealed that access difficulty to health services impacts both case detection probability and incidence rate. Minimal differences are recorded in the observed and estimated incidence of influenza cases considering social deprivation factors. In most years, areas in Victoria’s southwest and eastern parts have potential under-reported cases consistent with their relatively lower number of GP or GP clinics.

**Conclusion:**

The Bayesian model estimated a slight discrepancy between the estimated incidence and the observed cases of influenza in Victoria, Australia in 2013-2016 period. This is consistent with the relatively equal health resources and services in the state. This finding is beneficial in determining areas with potential under-reported cases and under-served health care. The proposed approach in this study provides insight into the impact of health inequality in disease detection without requiring costly and time-extensive surveys and relying mainly on the data at hand. Furthermore, the application of Bayesian model averaging provided a flexible modelling framework that allows covariates to move between case detection and incidence models.

## Background

Measuring health inequality is crucial as it reflects the performance of the country’s health system, which is crucial towards the healthy development of individuals, families and society as a whole [1]. Australia has an extensive health prevention and promotion plan in keeping all healthy as long as possible. According to the Australian Institute of Health and Welfare, it is essential to have access to timely, appropriate and quality health care at the right cost during ill health. In addition, all is not equal. Location, income, the existence of a disability, access to health services; all contribute to one’s health. The health system can be assessed by measuring how health care varies across geographic areas, the number of potentially preventable hospitalisations and adverse events in hospitals [2]. Given this, it overlays the importance of health care and disease studies to keep the nation healthy.

Recognising its importance, the World Health Organisation has been actively promoting to reduce the health inequalities within and across countries [1]. In the year 2000, Wilkinson and Symon [3] first measured the inequality of general practitioners in Australia. The study showed that non-metropolitan areas were relatively under-served of general practitioners. On the other hand, Hann and Gravelle [4] explored the same study on general practitioners in England and Wales from 1974 to 2003, and the findings showed increased maldistribution of general practitioners from mid-1980s to 2003. In Albania, Theodorakis et al. [5] found that there is a trend in the inequality of general practitioners while Toyabe [6] in Japan observed that the number of physicians in the hospital has increased significantly in urban areas but not in low population areas. Matsumoto et al. [7] assessed the distribution of primary care physicians in Japan and Britain to compare the primary care systems between the two countries. Results suggested that the distribution in Japan is less balanced than Britain’s by about twice the observed inequality [7].

Ohba et al. [8] compared the geographical inequalities of radiotherapy resources in two districts of Japan, Hokkaido-Tohoku and Tokyo. Results demonstrated that the inequality is higher in the Tokyo district. In China, Liu et al. [9] analysed the inequality of emergency medical services (EMS) in Chongqing city. This study aimed to determine the population and geographic distribution of EMS-related facilities and human resources. Findings suggested that there is a low allocation of resources and the provision of services did not meet the patients’ needs [9]. While Zhang et al. [10] studied the overall distribution of health resources and services in China as the country’s major goal is to achieve equity in the health system reform. It is observed that inequality is more apparent in the geographic distribution than per capita and better resources and services to the rich as compared to the poor, a risk for two-tiered health care system [10].

Disease studies and surveillance are important to minimise the burden of the disease that affects the health and quality of life. According to the Centers for Disease Control and Prevention (CDC), the end goal of disease surveillance is to keep people healthy. This can be done by detecting the disease when and where it happens, stopping before it spreads, studying the disease and improving prevention and control. According to CDC, spatial analysis of disease has crucial value in order to stop the spread and keep people healthy. Infectious diseases have a tricky nature of transmitting the virus in the community at a particular time and speed [11].

Influenza, being an epidemic infection, affects millions of people around the world on yearly basis [12]. The spread of a virus is very complex in local and global contexts, with many factors affecting its onset, time span, and severity. In 2018, Geoghegan et al. [13] conducted an in-depth spatio-temporal analysis of influenza in Australia driven by the study in the United States showing a strong geographical correlation. Given the complexity of the climate and a smaller population in Australia, albeit similar in land-size of the United States, this trend is yet unknown to apply in Australia. Results showed that despite the wide spatial and temporal variations, there is remarkable epidemiological synchronicity in the continent. This emphasises the importance of well-coordinated responses in times of outbreaks and human-to-human transmissions [13]. Moa et al. [12] explored Australia’s inter-seasonality of influenza wherein results indicated a relatively constant summer-to-winter influenza notifications ratio in the country, which is consistently supported by the constant yearly hospitalisation rates.

In terms of case detection, Shaweno et al. [14] used a Bayesian binomial mixture geospatial model to estimate tuberculosis (TB) incidence and case detection rate in Ethiopia. Stoner et al. [15] proposed a hierarchical Bayesian approach that uses prior information and corrects for under-reporting of the disease. They implemented their approach to analyse TB incidence and case detection in Brazil. These two studies provide us with a practical modelling framework to estimate the number of under-reported cases concerning other health services and socio-economic factors. With a very comprehensive study, Zipfel et al. [16] recently proposed a Bayesian framework to determine important factors on health disparities in influenza cases in the US. They consider socioeconomic covariates and transmission of the disease in relation to the socioeconomic status, and aim to capture spatial variations in health inequalities. They develop an epidemiological model and a spatial inferential model which is very similar to those of Shaweno et al. [14] and Stoner et al. [15]. Although Zipfel et al. [16] consider the spatial variability, they do not take the spatial correlation between adjacent areas into the modelling as Shaweno et al. [14] and Stoner et al. [15] did.

These studies show the importance of investigating two main health factors: the health resources and services and the disease concerning the geographical variation. In this study, we aim to extend the overall measure of inequality by exploring its impact on a granular level through Bayesian estimation for influenza cases in Victoria, Australia. As Zipfel et al. [16], we also consider socioeconomic factors through their spatial variation in our modelling. We utilise the estimates of under-reported cases due to the lack of health resources and services to infer spatial health inequality. To achieve this aim, we improve the case detection models developed for TB disease and implement them for influenza cases. In the Bayesian model frameworks of Shaweno et al. [14], Stoner et al. [15], and surveillance model of Zipfel et al. [16], the covariates belonging to the incidence rate and detection probability components of the model are determined by the practitioner before running the model. However, some of the socioeconomic covariates can influence either component of the model. Instead of assigning all the covariates to specific components, letting them float between the model components contributes to the performance of the model and captures the impact of floating covariates on both the incidence rate and case detection probability. We improve the modelling framework by introducing Bayesian model averaging (BMA) that allows flexibility in the covariates to move between detection probability and incidence rate components. In this way, we improve the modelling and fill in the gap by estimating the effect of health inequality on the disease detection rate, which does not only identify areas that have under-served health resources and services but also areas with possible under-reported disease cases as a result of this health inequality. The contributions of this study are that i) we use the concept of under-reporting to detect spatial health inequality considering the correlations between adjacent areas. ii) We utilise socioeconomic factors to model both incidence rate and detection probability without restricting them only to affect either the model’s case detection probability component or its incidence rate component. iii) We provide a picture of health inequality across the local government areas of Victoria, Australia in relation to influenza disease. Our approach in this study is readily generalisable to other countries and diseases to analyse spatial health inequality.

## Methods and data

This study will review the impact of access to health care on disease detection. Firstly, an overall inequality of health resources and services is measured using inequality indices. Secondly, we estimate the effect of this degree of inequality in detecting the disease using Bayesian estimation.

### Inequality measure

The Gini coefficient is a widely used inequality measure mainly applied in income and wealth. Several studies in the past have explored the distribution of health services from general practitioners to specialists and health resources such as emergency department and radiotherapy by using the Gini coefficient. Through this coefficient, these studies determined areas or groups of people experiencing inequality in the distribution. In Albania, Theodorakis et al. [5] used the Gini coefficient to compare the inequality of distribution of GPs in 2000-2004 and recorded a declining trend across the years. In Japan, Toyabe [6] compared the Gini coefficient with other inequality indices such Atkinson index and Theil index to assess the distribution of physicians where all the indices generated relatively the same findings. Also in Japan, Ohba et al. [8] determined that the inequality of radiotherapy health resources in Tokyo district is higher than in the Hokkaido-Tohoku by using the Gini coefficient. Furthermore, Matsumoto et al. [7] used Gini coefficient to validate that primary care physicians’ distribution in Britain is relatively equal than in Japan. On the other hand, in China, Liu et al. [9] used the Gini coefficient to determine that distribution of physicians and nurses in Chongqing city is relatively equal. In China as a whole, Zhang et al. [10] found that the geographic distribution of the health resources and health services is less equal than per capita using the Gini coefficient.

The value of Gini coefficient ranges from 0 to 1, wherein a lower Gini coefficient means a more equal distribution while a higher Gini coefficient means a more unequal distribution. In conjunction, the Lorenz curve is used to visualise the Gini coefficient. In this study, the application of these indices will determine the overall value and graphical representation of the inequality of health resources and services using Eq. (1) [17]:

1 $$\mathrm{G} = 1 - \sum\limits_{k=1}^{n} ({\mathrm{Y}_{k-1}} + {\mathrm{Y}_{k}})({\mathrm{X}_{k}} - {\mathrm{X}_{k-1}}),$$

where X_*k*_ is the cumulative proportion of the population for *k*=0,…,*n*, with X_0_=0, X_*n*_=1 and Y_*k*_ is the cumulative proportion of the health resources and services for *k*=0,…,*n* with Y_0_ = 0, Y_*n*_=1. The relationship between Gini coefficient and Lorenz curve is given in Eq. (2):

2 $$\mathrm{G} = \mathrm{A}/(\mathrm{A}+\mathrm{B}),$$

where A is the area above the curve and B is the area below the curve.

### Bayesian estimation

Shaweno et al. [14] introduced the use of the Bayesian technique in estimating tuberculosis incidence and case detection rate in Ethiopia. They used a two-component Bayesian model composed of a model for incidence rate and another model for the case detection rate. Their study was able to determine TB hotspots that were not previously identified. Further, the model used in their study clearly separated true incidence and case detection that enables to identify whether a high notification rate is due to a failing health system, failing to detect patients rapidly or an efficient health system diagnosing all incidences quickly. Results revealed a vast difference between notification rate and estimated incidence rate in areas with no health facilities, highlighting the importance of reducing the number of missed cases. This approach provided an alternative and practical approach to estimate incidence by only using routinely collected surveillance data [14]. Stoner et al. [15] developed a similar approach by correcting the under-reporting in count data using enhanced models and additional prior information to supplement partial information in the data. In their study, the model for incidence includes various social deprivation covariates such as homelessness and incarceration as some spatial variability can be attributed to these covariates [15]. These approaches from Shaweno et al. and Stoner et al. are used in this study to estimate the effect of unequal health resources and services in disease detection, taking into consideration the spatio-temporal diversity and the correlation between neighbouring areas.

Under the assumption that individual cases are detected independently at a fixed rate and are conditional on individual incidence [14, 15], two main component models to capture (true) incidence and case detection probability are explored in this study using the following modelling framework:

3 $$\text{Incidence}_{t,s} \sim{} \text{Poisson}({\lambda_{t,s}}),$$

such that,

4 $$\log({\lambda_{t,s}}) = \alpha_{0} \; + \sum\limits_{k=1}^{K} \; \alpha_{k} \; \mathrm{u}^{(k)}_{t,s},$$

where {*u*^(*k*)^} includes covariates that give rise to the number of cases including balancing effect such as population counts. The expected number of detected cases, Z_*t,s*_, follows a binomial distribution conditional on the hidden true incidence rate and the probability of the case being detected:

5 $$\mathrm{Z}_{t,s} \sim{} \text{Binomial} (\pi_{s}, \text{Incidence}_{t,s}),$$

where Incidence_*t,s*_ is defined in Eq. (3) and

6 $${\log\left(\frac{\pi_{s}}{1-\pi_{s}} \right)} = {\beta_{0} + \sum\limits_{j=1}^{J} \beta_{j} \;\mathrm{v}^{(j)}_{t,s}.}$$

In Eq. (6), {v^(*j*)^} is the vector of covariates related to the disease detection process. This allows to record the severity of under-reporting and what it pertains to. Using integration and Bayes’ rule [15],

7 $$\text{Incidence}_{t,s} - \; \mathrm{Z}_{t,s} \sim{} \text{Poisson} ((1-\pi_{s}) \lambda_{t,s}),$$

where *s* represents the space index and *t* the time index to capture both the spatial and temporal effects.

One of the risks highlighted by Stoner et al. [15] is the uncertainty in identifying or classifying whether a covariate belongs to the case detection process or the true incidence; hence, the distinction between if a covariate impacts case detection rate or incidence rate is unclear. We call this type of covariate *floating covariate* in this study. To overcome this ambiguity, we propose to use Bayesian model averaging (BMA) within the implementation of the modelling framework of Stoner et al. [15]. We establish a model space *℘*(*S*) such that all elements are considered to estimate the parameter of interest *Δ* given the data D [18]. Then, the posterior distribution of the parameter of interest is calculated as in Eq. (8):

8 $$P(\Delta|D) = \sum\limits_{m \in \wp(S)} P(\Delta|m, D)P(m|D).$$

BMA allows all the alternative models in a model space *℘*(*S*) to have a chance to impact the overall parameter estimates proportional to their posterior model probabilities that are derived by

9 $$P(m|D) = \frac{P(D|m)P(m)} {{\sum\nolimits}_{m' \in \wp(S)} P(D|m')P(m')}.$$

In this study, a model space is created by considering floating covariate(s) in both of case detection probability and the incidence rate components of the overall model, and we let the BMA capture the part where the floating covariate will most likely be. This is our main contribution over the existing work of Shaweno et al. [14] and Stoner et al. [15].

### Data and descriptive analysis

The method will be applied using the notified cases of influenza in Victoria, Australia. The notified cases of influenza are requested from National Notifiable Diseases Surveillance System from 2013-2016 spatially aggregated in 79 Local Government Areas (LGA) [19]. The data for Victoria, which contains the social deprivation covariates including the availability of health resources and services and the access difficulty covariate, are all accessed from the Australian Urban Research Infrastructure Network (AURIN) Portal [20, 21]. The estimated resident population is sourced from the Australian Bureau of Statistics [22]. The influenza cases and all other covariates were aggregated by LGA and year to evaluate the spatial and temporal effects.

**Fig. 1:**
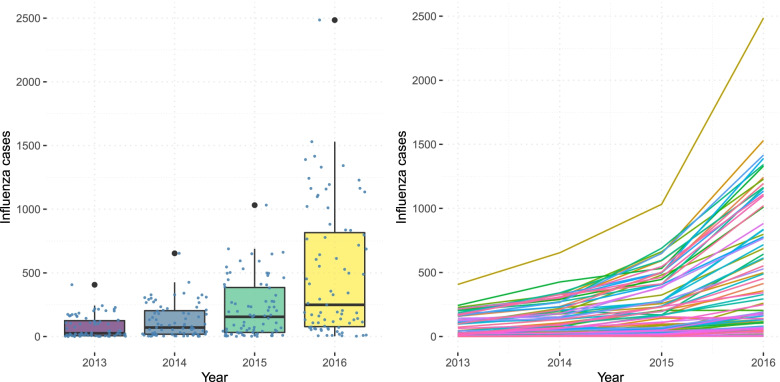
Distribution of Influenza Notifications 2013-2016. Each line on the right panel corresponds to an LGA

The distribution of influenza notifications and their trends by LGA over the observation period of 2013-2016 are given in Fig. 1.

The right panel of Fig. 1 shows the yearly distribution of the notified cases of influenza regardless of type in Victoria from 2013-2016. The number of cases across the years has been substantially increasing with an increasing variation. Consequently, this variation may cause more variable estimates to arise during Bayesian estimation. The number of influenza cases is plotted across time for each LGA of Victoria on the left panel of Fig. 1. There is a quadratic increase in the number of influenza cases in the LGAs of Victoria. Based on this observation, we introduce a quadratic function of time into the model.

**Fig. 2:**
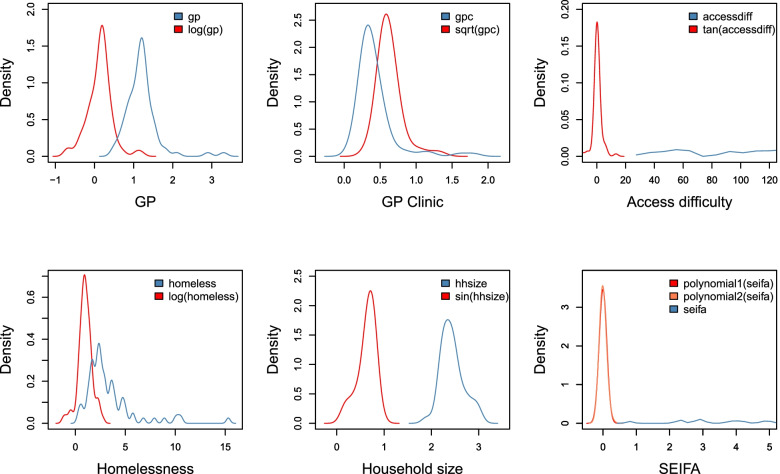
Exploratory analysis of the raw and transformed covariates

**Fig. 3:**
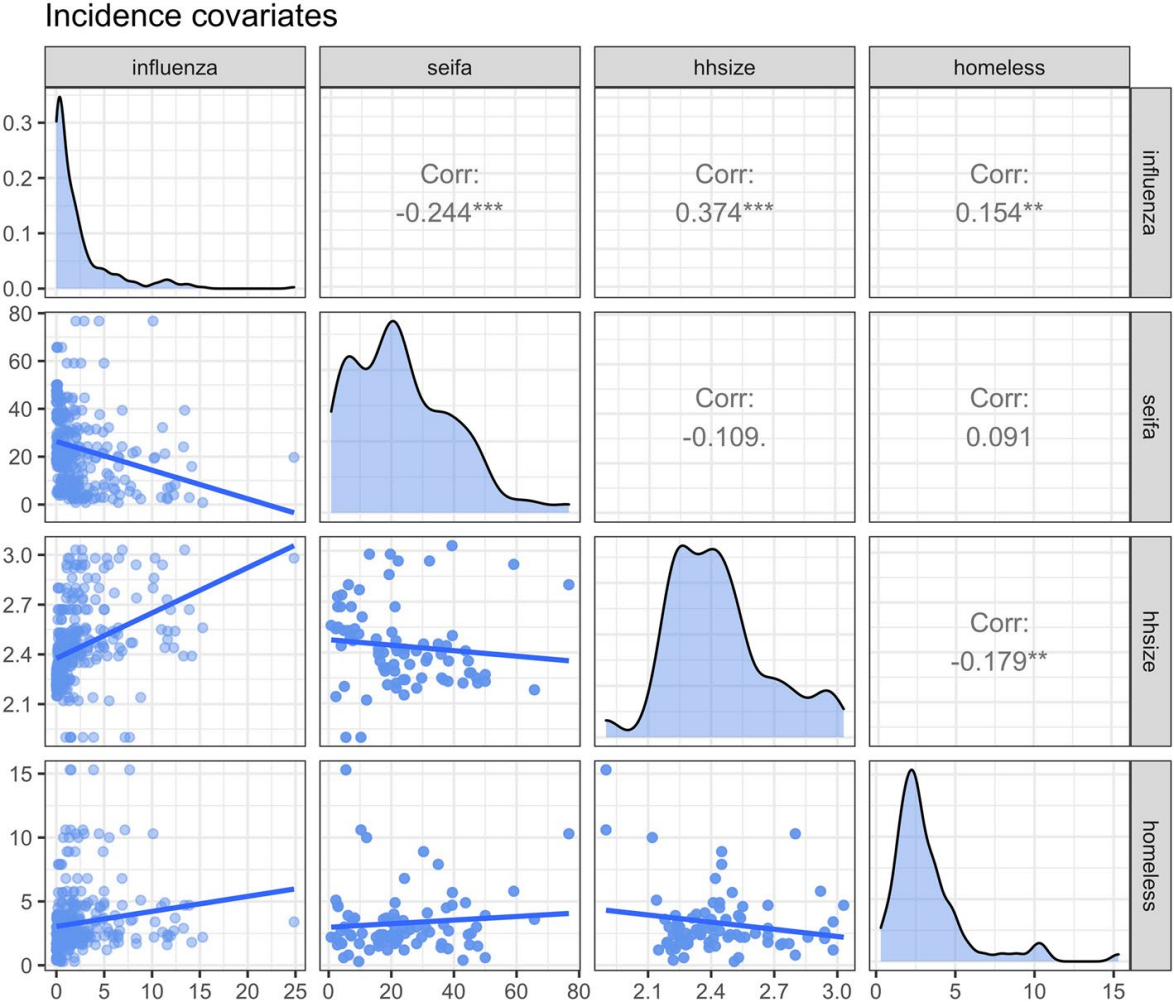
Correlation matrix of the incidence covariates. Influenza counts are divided by 100 for plotting purposes. *: *P*-value <0.05, **: *P*-value <0.01, ***: *P*-value <0.001

Figure 2 shows density plots of the covariates considered in the models and Fig. 3 shows the correlation of the incidence covariates against the influenza cases. For the incidence model in Eq. (10), the included covariates are population, socio-economic indexes for areas (SEIFA), household size, homelessness and year as the temporal lag. With various social factors incorporated in deriving SEIFA, it is transformed to an orthogonal polynomial with a degree of 2 to minimise multi-collinearity since it may interact with other social factors in our model, as also suggested by Stoner et al. ([15], see page 1484). From Fig. 3, there is a significant and negative correlation between the influenza cases and SEIFA, and significant positive correlations between influenza cases and household size and homelessness. For the case detection probability model in Eq. (11), GP and GP clinic per 1,000 population are considered. Further, the floating covariate, access difficulty, is to be explored in both models. Since Markov Chain Monte Carlo (MCMC) methods iteratively optimise the model parameters [23], it is time-consuming to get representative and accurate chains that do not have significant autocorrelation when the covariates’ distributions have a large range and are skewed with outliers. When there are outliers in the covariates, they have the potential to make the parameter structure highly correlated and reduce the speed of convergence ([24], see page 177). In order to shorten the tails of the covariate’s distributions and move outliers towards the centre of their distributions, different transformations, including trigonometric transformations and the family of Box-Cox transformations that includes log, reciprocal, square root, and polynomial transformations, are considered. The transformation that reduces the range of each covariate’s distribution and makes it most symmetric is implemented. For this aim, adopting suitable transformations is considered. Common transformations include log, reciprocal, square root, in general, the family of Box-Cox transformations, and trigonometric transformations ([25], see page 169). Standardisation by centring and scaling, Box-Cox transformations by optimising the power parameter, and basic trigonometric transformations such as sine, cosine, and tangent are applied for GP, GP clinic, Access difficulty, Homelessness and household size covariates. The transformation that reduces the range of each covariate’s distribution and makes it most symmetric is implemented. Red density plots in Fig. 2 show the transformed versions of the covariates.

### Bayesian modelling and model space

We apply our Bayesian framework using the dataset to capture (true) incidence and case detection probability in Eqs. (3)-(7). For the incidence rate component in Eq. (3), we set the model in Eq. (10):

10 $$\begin{array}{rl} \log({\lambda_{t,s}}) =& \log(\text{Pop}_{t,s}) + \; \alpha_{0} \; + \boldsymbol{\alpha}_{\boldsymbol{1}} \; \text{seifa}_{s} + \alpha_{2} \; \text{hhsize}_{s} \; + \alpha_{3} \; \text{homeless}_{s} + \; \phi_{s} \; + \; \theta_{s} \\ &+ \; f(t) + \; I(\cdot)\alpha_{4}\;\text{AccessDifficulty}_{s}, \end{array}$$

where ***α***_***1***_=(*α*_1.1_,*α*_1.2_) show the parameters resulting from the polynomial transformation of SEIFA, *f*(*t*) is a quadratic function of time defined as *f*(*t*)=*α*_5_*t* + *α*_6_*t*^2^ and the presence of the floating uncategorised covariate AccessDifficulty_*s*_ is controlled by the indicator function *I*(·) (in Eq. (11) as well). The expected number of detected cases, Z_*t,s*_, given the distribution of the health resources and services follows a binomial distribution conditional on the hidden true incidence rate and the probability of the case being detected as given in Eq. (5) where we define Incidence_*t,s*_ as in Eqs. (3) and (10), and set the model in Eq. (11) for the case detection probability:

11 $${\log\left(\frac{\pi_{s}}{1-\pi_{s}} \right)} = {\beta_{0} + \beta_{1} \; \text{GP}_{s} + \beta_{2} \;\text{GPclinic}_{s} + I(\cdot)\beta_{3}\; \text{AccessDifficulty}_{s},}$$

where space index *s* represents the LGAs in Victoria *s*=0,1,…,79 and *t* and the time index *t*=1,2,3,4 represents years 2013-2016, respectively.

The incidence model in Eq. (10), represented by Poisson distributed *λ*_*t,s*_, is modelled using the population, SEIFA, homelessness and household size covariates. These covariates were considered in relation to our descriptive analysis and the study by Stoner et al. [15] on tuberculosis for social deprivation factors given the similar infectious nature of the disease. In Eq. (10), the log(Pop_*t,s*_) term incorporates the varying population, seifa_*s*_ is the index of relative socio-economic disadvantage (IRSD), hhsize_*s*_ is the average household size and homeless_*s*_ is the estimated number of homeless people per 1,000 population varying in space and time. *ϕ*_*s*_ represents the spatially structured effect modelled as an intrinsic Gaussian autoregressive model [26] to capture the effects of contiguous areas wherein areas are contiguous when *s*^′^≠*s* shares a common geographical boundary. *θ*_*s*_ is the spatially unstructured random effect. The function of time *f*(*t*) is implemented in a quadratic form to emphasize the substantial increase in influenza cases through the years as shown in Fig. 1.

The case detection probability in Eq. (11), represented by the logit transformed *π*_*s*_, is modelled using GP and GP clinic covariates. These covariates were considered noting that the data compiled in the NNDSS are greatly influenced by patients who seek healthcare [27]. In Eq. (11), the term GP_*s*_ is the distribution of general practitioner per 1,000 population and GPclinic_*s*_ is the distribution of general practitioner clinic per 1,000 population. The floating covariate AccessDifficulty_*s*_ is the estimated number of people 18 years old and over who experienced difficulty in accessing healthcare in the last 12 months when needed, with a major reason being the cost of service. This is the floating covariate of our study for the reason that it has potential to affect both the case detection and incidence models. Therefore, it is left unclassified whether to belong in the case detection probability or the incidence rate.

There is also the possibility that the sub-models of the models in Eqs. (10) and (11) perform better than the full models. Therefore, the sub-models are also considered alongside the full models at the model averaging stage. To reflect this, 24 candidate models are formulated in the model space of our study. Figure 10 of Appendix A shows the 24 models considered in the model space *℘*(*S*) the study. These models are combinations of the individual case detection probability and incidence models. The floating covariate AccessDifficulty is considered under the incidence rate component in Models 1-12, while it appears under the case detection probability component in Models 13-24. By this setting for AccessDifficulty, we do not need to decide on the place of AccessDifficulty covariate before running the Bayesian model. Instead, the information in the data designates the most suitable part of the model where AccessDifficulty belongs.

The model diagrams for the implementation are presented in Figs. 11 and 12 of Appendix A, respectively, for single model implementation and Bayesian model averaging. In Fig. 11 of Appendix A, non-informative prior and hyper-prior distributions are induced on all coefficients of the models in Eqs. (10) and (11), given the absence of prior information about model parameters. The vector of residual spatial variation parameters ***ϕ*** has a priori conditional autoregressive Gaussian process (CAR) [28] with zero men vector and covariance matrix of [*τ*(*D*−*W*)]^−1^, where *τ* controls precision, *W* is the adjacency matrix and *D* is the diagonal matrix with the sum of the adjacent neighbours for each LGA. The CAR process is to capture the spatial dependence between irregularly spaced sites. For the spatially unstructured random effect parameter *θ*_*s*_, a non-informative normal prior with normal and half-normal hyper-priors on its parameters are set to induce prior variability to improve the accuracy of the generated chains during MCMC implementation. Figure 12 of Appendix A represents the Bayesian model averaging through a non-informative categorical prior distribution that assigns an equal prior probability to each model in the model space.

### Model evaluation and implementation

The diagnostics of the models are evaluated as a preliminary step to determine the representativeness and accuracy of the MCMC samples. Moreso, a posterior predictive model checking is conducted to assess that the model fits the observed data.

The data are mainly processed and modelled in R software version 3.6.0 using various required packages. The final processed data are used to calculate the Gini coefficient and visualise the Lorenz curve. The Bayesian models are fitted using MCMC in JAGS; leveraging its capability to perform model averaging. In addition, the module GeoJAGS is used to generate structured spatial effect of the model through the CAR process [29].

The implementation of the proposed modelling framework in JAGS provides the opportunity to do model averaging which was not implemented in the previous studies that used WinBUGS and Nimble [14, 15]. However, it is noted that the usage of the module GEOJAGS in JAGS to run the complex spatial model such as ICAR is still at its early stage as compared to WinBUGS and Nimble [29].

The 24 models listed in Fig. 10 of Appendix A ran in JAGS with 140,000 iterations, 2 chains, 19 thinning while discarding the first 200,000 iterations as burn-in to configure the most probable models to represent the data.

## Results

**Fig. 4:**
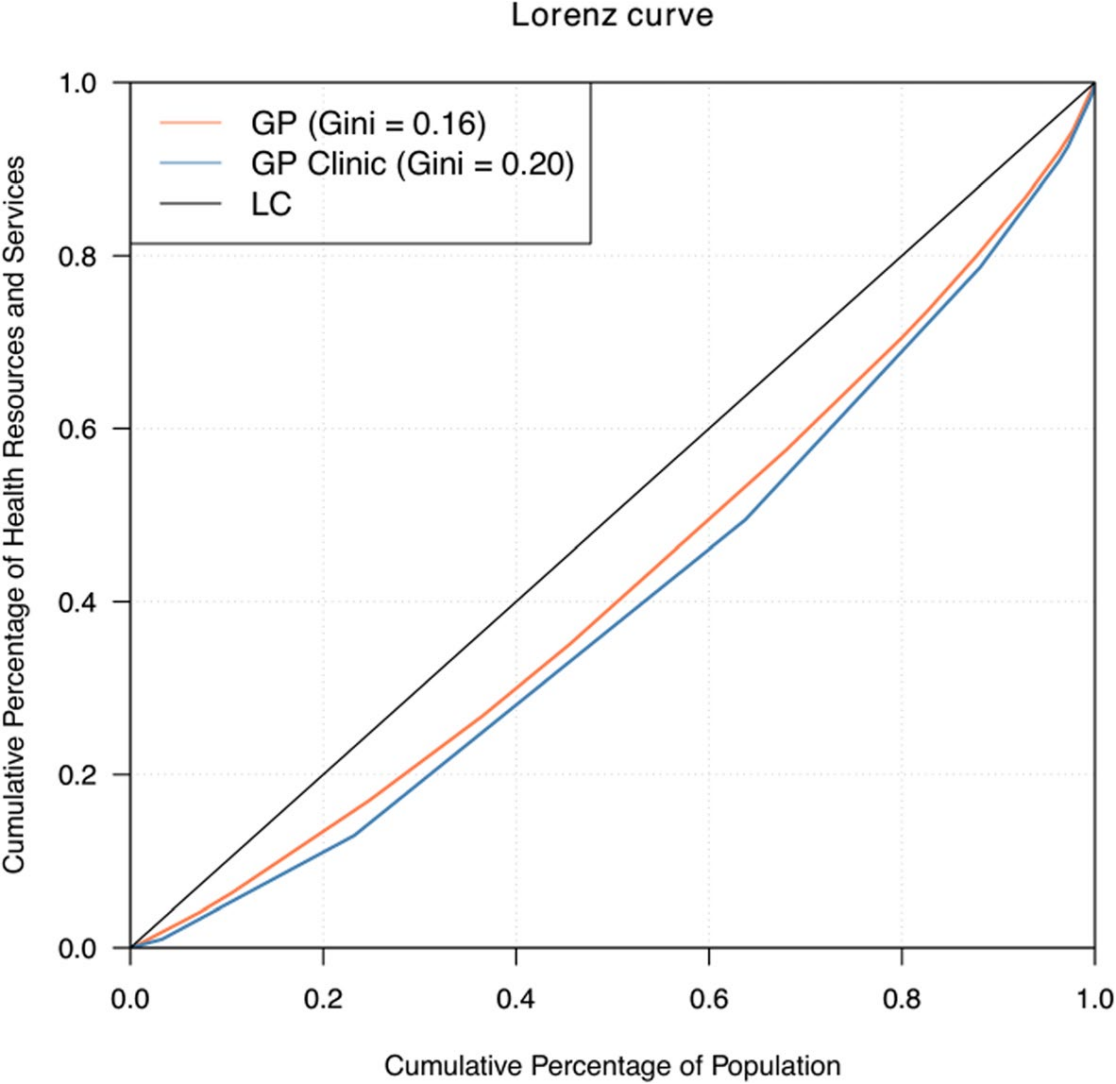
Distribution of GP and GP clinic in Victoria 2013-2016

Figure 4 shows the inequality of the number of GP and GP clinics across LGAs in Victoria and the corresponding Gini coefficient. The closer the curve is to the line of perfect equality, the more equal the health resources and services are across areas. Overall, the distributions of both numbers of GP and GP clinics in Victoria are relatively equal, wherein 80 percent of the population shares roughly 70 percent of GP (Gini = 0.16) and GP clinics (Gini = 0.20). Further, the number of GPs is slightly more equal as compared to GP clinics in the population.

**Fig. 5:**
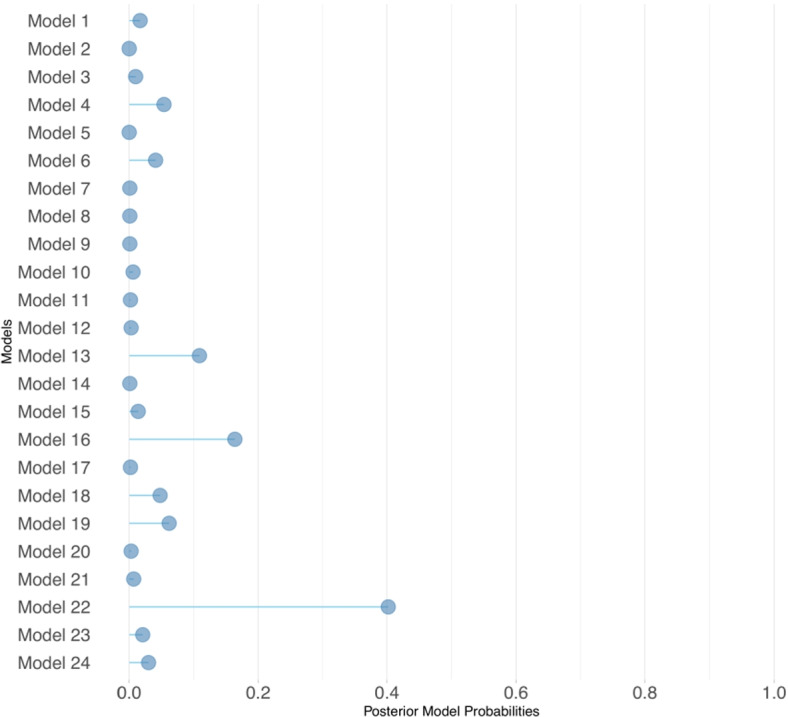
Posterior model probabilities

Figure 5 shows the posterior model probabilities of all the models in the model space, and Table 1 gives the top 5 models in terms of posterior model probability along with their model equations. In Fig. 5, the 24 models show a competitive contribution to the predictions with the domination of Model 22, dominating almost half of the model visits during MCMC.

**Table 1:** 5 Models streamlined during model selection

Model No.	Combination of *λ*_*t,s*_ *and* *π*_*s*_	*P*(*m*|*z*)
Model 4	$$\begin {array}{rl} \lambda _{t,s} \; = \log (\text {Pop}_{s}) \; & + \; \alpha _{0} \; + \alpha _{1.1} \; \text {seifa}_{s} + \alpha _{1.2} \; \text {seifa}_{s} + \alpha _{2} \; \text {hhsize}_{s}\\ & + \; \alpha _{4} \; \text {AccessDifficulty}_{s} + \; \phi _{s} \; + \; \theta _{s} \; + \; f(t) \end {array}$$	0.054
	*π*_*s*_ =*β*_0_+*β*_1_ GP_*s*_+*β*_2_ GPclinic_*s*_	
Model 13	$$\begin {array}{rl} \lambda _{t,s} \; = \log (\text {Pop}_{s}) \; & + \; \alpha _{0} \; + \alpha _{1.1} \; \text {seifa}_{s} + \alpha _{1.2} \; \text {seifa}_{s} + \alpha _{2} \; \text {hhsize}_{s} \\ &+ \; \alpha _{3} \; \text {homeless}_{s} + \; \phi _{s} \; + \; \theta _{s} \; + \; f(t) \end {array}$$	0.109
	*π*_*s*_ =*β*_0_+*β*_1_ GP_*s*_+*β*_2_ GPclinic_*s*_+*β*_3_ AccessDifficulty_*s*_	
Model 16	$$\begin {array}{rl} \lambda _{t,s} \; = \log (\text {Pop}_{s}) \; & + \; \alpha _{0} \; + \alpha _{1.1} \; \text {seifa}_{s} + \alpha _{1.2} \; \text {seifa}_{s} + \alpha _{2} \; \text {hhsize}_{s} \\ & + \; \phi _{s} \; + \; \theta _{s} \; + \; f(t) \end {array}$$	0.164
	*π*_*s*_ =*β*_0_+*β*_1_ GP_*s*_+*β*_2_ GPclinic_*s*_+*β*_3_ AccessDifficulty_*s*_	
Model 19	$$\begin {array}{rl} \lambda _{t,s} \; = \log (\text {Pop}_{s}) \; & + \; \alpha _{0} \; + \alpha _{1.1} \; \text {seifa}_{s} + \alpha _{1.2} \; \text {seifa}_{s} \\ & + \; \alpha _{3} \; \text {homeless}_{s} + \; \phi _{s} \; + \; \theta _{s} \; + \; f(t) \end {array}$$	0.062
	*π*_*s*_ =*β*_0_+*β*_1_ GP_*s*_+*β*_2_ GPclinic_*s*_+*β*_3_ AccessDifficulty_*s*_	
Model 22	$$\begin {array}{rl} \lambda _{t,s} \; = \log (\text {Pop}_{t,s}) \; & + \; \alpha _{0} \; + \alpha _{1.1} \; \text {seifa}_{s} + \alpha _{1.2} \; \text {seifa}_{s} \\ & + \; \phi _{s} \; + \; \theta _{s} \; + \; f(t) \end {array}$$	0.402
	*π*_*s*_ =*β*_0_+*β*_1_ GP_*s*_+*β*_2_ GPclinic_*s*_+*β*_3_ AccessDifficulty_*s*_	

Four out of the top five models include access difficulty in the detection probability component. However, in Model 4, the access difficulty covariate contributes to the incidence rate component. This result implies that the access difficulty covariate should neither be classified under the case detection probability nor the incidence model. Both components seem to benefit from the effect of the access difficulty covariate. Looking further, in the top 5 models in Table1, Model 4 and Model 16 are model counterparts with access difficulty being swapped between the incidence rate and case detection probability components of the model. Apparently, the use of BMA provides a more inclusive prediction by taking the contribution of all the models that have a non-zero weight and eliminating the need for a pre-modelling decision for fixed covariate categorization.

**Fig. 6:**
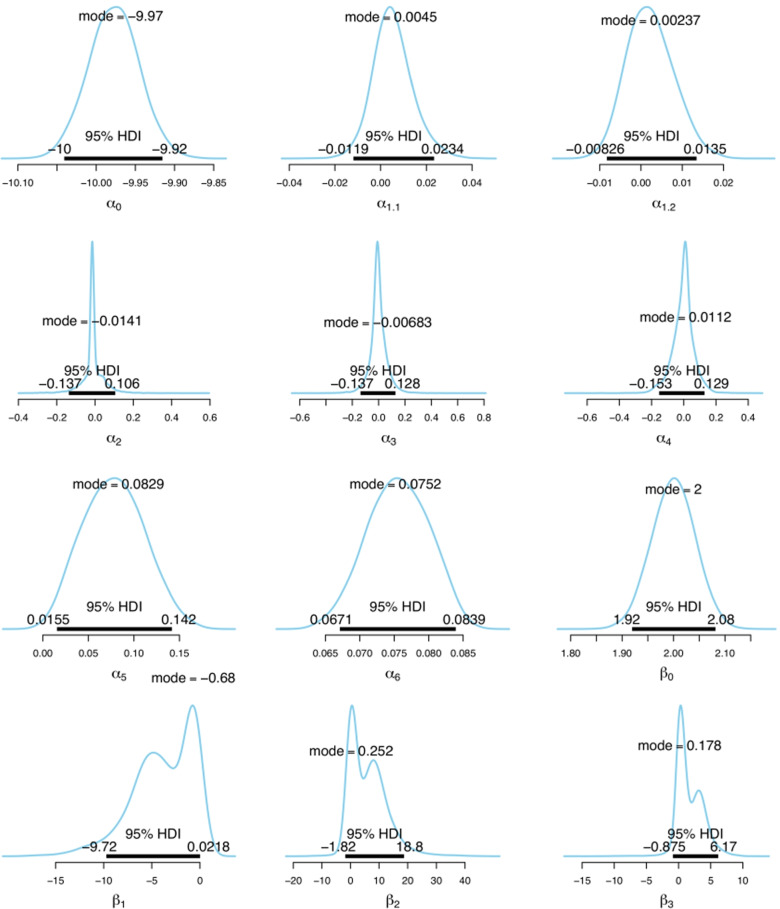
Predictors of incidence and case detection rate

Figure 6 shows the posterior distributions of model parameters. The parameter estimates of *β*_1_,*β*_2_ and *β*_3_ in *π*_*s*_ component which has health resources and resources predictors, have bi-modality. For *β*_3_, when access difficulty is not in the *π*_*s*_ component, the realisations of this coefficient come from the prior distribution centred around zero. When it is in the *π*_*s*_ component, the realisations are created by the posterior distribution. As a result, we have a bi-modal posterior for this coefficient. For *β*_1_ and *β*_2_, the reason for having bi-modal posteriors is the variability caused by the inclusion of the access difficulty in half of the 24 models. This also depicts varying efficiency in case of detection rates among GP and GP clinics across LGAs regardless of the number of available health resources and services or telehealth services, which can create cross-border detection.

### Incidence and case detection rates

**Fig. 7:**
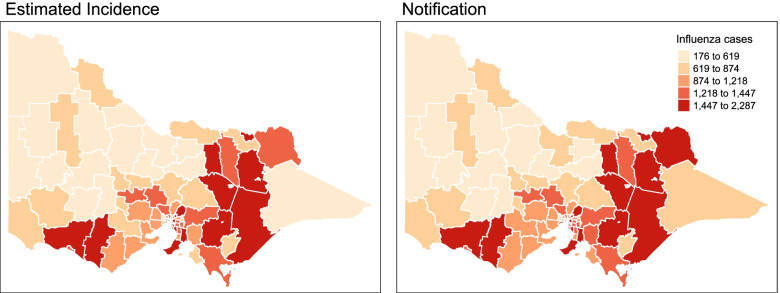
Estimated influenza incidence and notification in Victoria 2013-2016 (per 100,000 population)

**Fig. 8:**
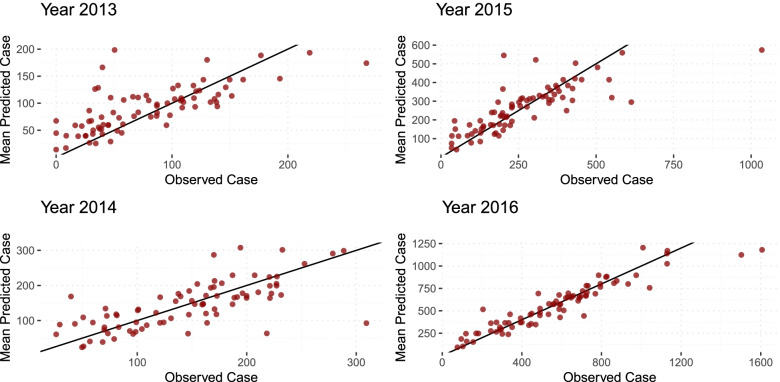
Observed and predicted influenza cases (per 100,000 population) 2013-2016

**Fig. 9:**
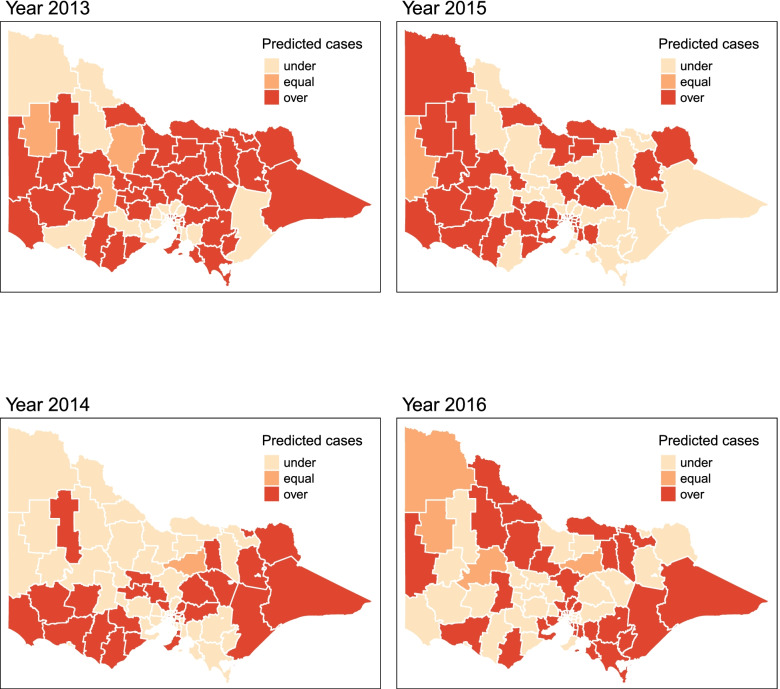
Predictions of under and over-reported influenza cases across the LGAs of Victoria, Australia for 2013-2016

**Fig. 10:**
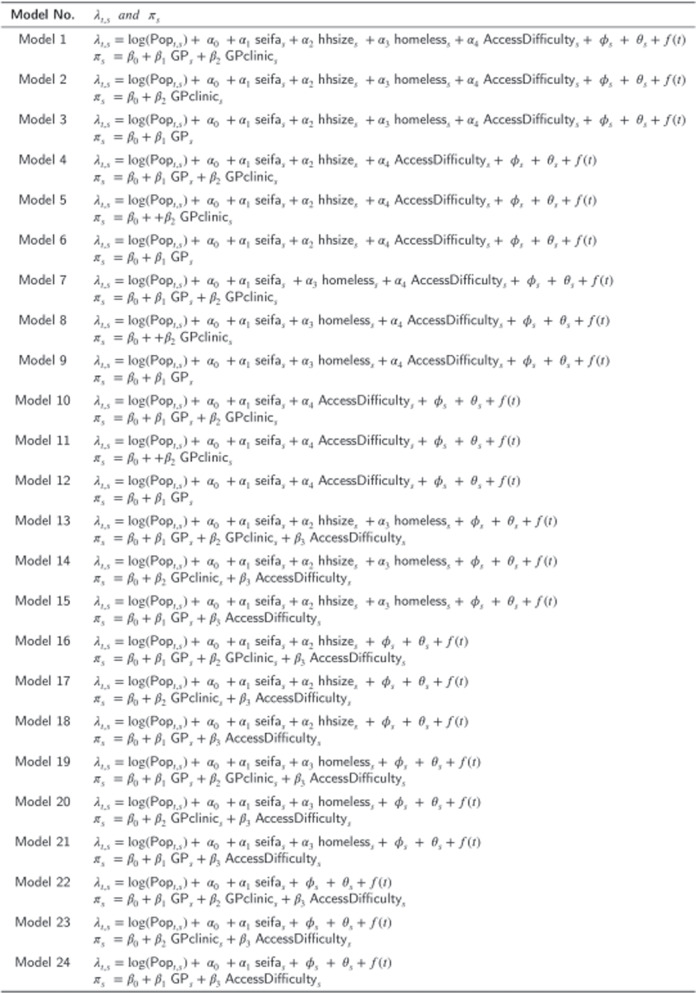
24 Candidate Models. Here *f*(*t*)=*α*_5_*t*+*α*_6_*t*^2^

**Fig. 11:**
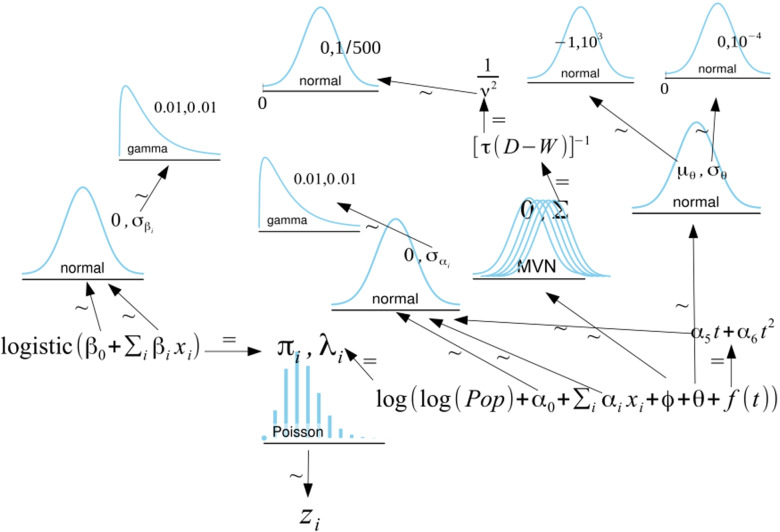
Model Diagram

**Fig. 12:**
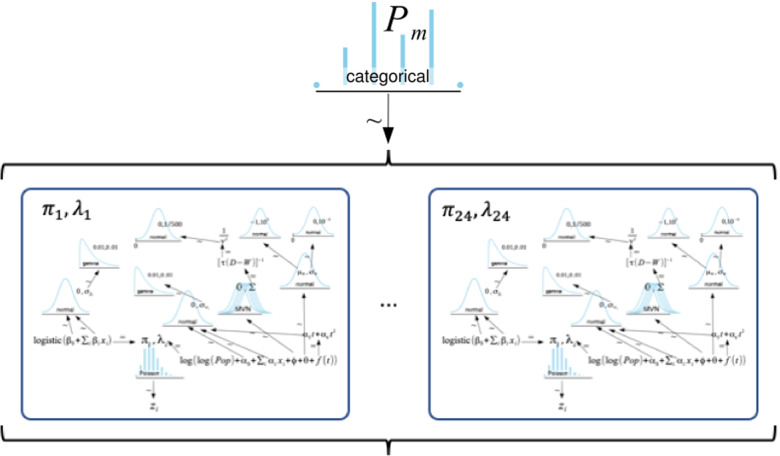
Bayesian Model Averaging (BMA)

Figure 7 shows the comparison maps of the estimated incidence and observed notification of influenza in Victoria in 2013-2016, wherein the darker the colour depicts a higher number of influenza cases. Figures 8 and 9 show the comparison of predicted and observed influenza cases by year and LGA. For Fig. 9, the dark red colour means the predicted number of influenza cases in the area is higher than the observed number of cases. This indicates the possibility of having under-reported cases for that specific area.

From Fig. 7, we observe that the estimated incidence and actual notifications of influenza are correlated with highly recorded cases in the southern and eastern LGAs of the state. Further, the differences are very minimal. This depicts a good detection process among LGAs in Victoria considering the relatively equal distribution of health resources and services. Figure 7 shows the validity of the models used given the close fit of the predictions and actual notifications.

In Fig. 8, looking closely at the predicted cases in comparison to the observed cases of influenza by year, it is apparent that increasing case detection rate is observed through the years. Reviewing the potential LGAs with under-reported cases by year in Fig. 9, a changing pattern is recorded. In 2013, most of the LGAs were deemed to have under-reported cases, and 2014 seems to concentrate at the southwest and eastern parts, moving towards a more spread out under-reported cases in 2015-2016. Overall, the number of GP and GP clinics in the dataset from 2013-2016 is constant. The changing pattern in the number of cases detected across areas and years depicts either a generally increasing number of influenza cases or improvement in the case detection process in the state. Performing a more detailed look into the potential under-reported areas where consistently observed in most years in Fig. 9, potential areas for under reported cases in the southwest are Glenelg, Southern Grampians, Ararat, Moyne, Corangamite, Colac-Otway, Surf Coast and eastern such as Alpine, Towong and East Gippsland. These areas are recorded accordingly to have relatively lower the number of GP or GP clinic.

## Discussion

This study demonstrated the impact of the degree of inequality in health resources and services into disease detection using two-component Bayesian modelling. Using an inequality measure in conjunction with Bayesian estimation provides a picture of the severity of under-served areas in terms of access to health resources and services in addition to its efficiency to detect disease cases as it happens by accounting for existing social factors. The Bayesian framework proposed in this study is a practical approach that does not require time-intensive and costly surveying of detailed geography levels such as LGA and expert opinion on disease rates, though acknowledges their importance. In addition, it provides an estimation approach that relies mainly on the observed data at hand.

In line with the relatively equal number of GP and GP clinics (with Gini coefficients of 0.16 and 0.20, respectively) across the LGAs in Victoria, Australia in 2013-2016, the estimated incidence of the influenza cases showed minimal discrepancy with the observed influenza cases. This result takes into account the social deprivation factors that can have an impact on the actual count of influenza incidence. Through the years, there has been an increasing trend in the number of influenza cases in the state. Looking closely at the areas, areas in eastern and southwestern parts of Victoria recorded potential under-reporting in most years. These areas accordingly are recorded to have either lower number of GP or GP clinic. This result depicts where the potential increase is happening vis-à-vis availability of health resources, services, and other social factors which can provide insight into areas with the possibility of an outbreak. Moreover, it identifies not only possible areas with a relatively lower number of GP and GP clinics that are unable to detect cases immediately, but also it explores the increase in estimated incidence rate that can happen due to social deprivation factors such as homelessness and higher index of relative socio-economic disadvantage in an area and its neighbouring areas.

Use of model averaging in this study, which is an improvement over the studies of Shaweno et al. [14] and Stoner et al. [15], helped to determine the classification of the floating or uncategorised covariate access difficulty. This covariate can be seen to impact both the case detection probability and the incidence rate of influenza. Bayesian model averaging provided a model framework that lets a covariate influence both model components without implementing fixed covariate categorisation. After running our Bayesian framework with 24 candidate models, the access difficulty was neither classified as a fixed covariate into the case detection probability component nor the incidence rate component. The posterior model probabilities suggest that all models have a contribution, and there is no need for the selection of one particular model or a fixed covariate categorisation. This finding provides an alternative and flexible modelling framework when any of the covariates seem to have overlapping effects between the case detection and incidence models.

## Conclusion

This study showed the connection of health inequality into a crucial matter of disease detection. The estimation of the impact of health inequality in terms of problems in disease detection can encourage policymakers to actively pursue equal access, especially in under-served areas and prevention in the spread of disease in the areas with potentially under-reported cases. Further, this practical and timely modelling using Bayesian estimation provides an alternative contemporary approach, especially during time-restricted situations such as an outbreak or unexpected surge of disease cases since it does not require costly and time-consuming surveying and preparations.

This hand-in-hand approach of using an inequality measure and Bayesian estimation mainly helped to identify finer level of areas with under-served health resources and services in addition to areas with possible under-reported disease cases. Moreover, by using two-component modelling Bayesian estimation for case detection probability and incidence rate, it distinguishes effects coming from the inequality of health resources and services or other social deprivation factors.

Lastly, this study demonstrated the use of Bayesian model averaging to enable researchers to eliminate the need for covariate categorisation, especially with overlapping covariate effects to both incidence and case detection models. The use of Bayesian model averaging provides the inclusion of all candidate Bayesian models’ effects to the predictions and allows the models to find their chance during implementation.

To note some limitations of this study, the time frame of this cross-sectional data analysis is only 2013-2016. Due to the five-year interval of the census data collection, some covariates are not observed for each year; hence, the same information is repeated across the years of study. Nonetheless, as a future study, exploring the application of our Bayesian modelling approach to other types of diseases and the inclusion of other disease-specific covariates can be considered. It would be helpful to explore the impact of various degrees of health inequality on the disease detection estimates. Doing so can provide ideal and achievable resource allocation without compromising the delivery of an efficient disease detection process.

## Data Availability

The datasets are available in the Australian Urban Urban Research Infrastructure Network (AURIN) Portal and the National Notifiable Disease Surveillance System (NNDSS) (available upon request).

## Appendix A: additional figures

Figure A1 lists the candidate models that are implemented in the study. Figure A2 shows the diagram of the Bayesian model, and Figure A3 outlines the proposed Bayesian model averaging approach.

## Abbreviations

AIHW: Australian Institute of Health and Welfare
AURIN: Australian Urban Research Infrastructure Network
BMA: Bayesian Model Averaging
CDC: Centers for Disease Control and Prevention
GP: General Practitioner
IRSD: Index of Relative Socio-economic Disadvantage
JAGS: Just Another Gibbs Sampler
LGA: Local Government Area
MCMC: Markov Chain Monte Carlo
PCP: Primary Care Physicians
SEIFA: Socio-Economic Indexes for Areas
WHO: World Health Organisation
